# Human Muscle-Derived Cells Injected into Acute and Chronic Muscle Injuries Engraft and Support Regenerative Markers

**DOI:** 10.3390/bioengineering13091022

**Published:** 2026-09-02

**Authors:** Aislin M. West, Jonathan T. Dillon, Kevin J. Hart, Seth T. Kreger, Nicholas J. Amoroso, Zvi Schwartz, David J. Cohen, Michael J. McClure

**Affiliations:** 1 Department of Biomedical Engineering, College of Engineering, Virginia Commonwealth University, Richmond, VA 23284-3068, USA; westam8@vcu.edu (A.M.W.); dillonj4@vcu.edu (J.T.D.); zschwartz@vcu.edu (Z.S.); djcohen@vcu.edu (D.J.C.); 2 Cook MyoSite, Pittsburgh, PA 15238-2806, USA; kevin.hart@cookmyosite.com (K.J.H.); seth.kreger@cookmyosite.com (S.T.K.); nicholas.amoroso@cookmyosite.com (N.J.A.); 3 Department of Periodontics, University of Texas Health Science Center at San Antonio, San Antonio, TX 78229, USA; 4 Department of Orthopedic Surgery, School of Medicine, Virginia Commonwealth University, Richmond, VA 23284, USA

**Keywords:** urinary incontinence, skeletal muscle, regeneration, muscle-derived cells, muscle injury

## Abstract

Urinary incontinence (UI), the involuntary leakage of urine, is a common condition that imposes significant physical, emotional, and financial burdens. Injury to the external urethral sphincter (EUS) contributes to myogenic UI by damaging the skeletal muscle responsible for urinary control. This study evaluated the engraftment and therapeutic potential of human muscle-derived cells (hMDCs) using acute and chronic skeletal muscle injury models. Immunocompromised female rats received cardiotoxin (CTX) or volumetric muscle loss (VML) injuries to the tibialis anterior muscle. Animals were treated with 100,000 or 500,000 passage 3 or passage 6 hMDCs injected one day after CTX injury or six weeks after VML injury. VivoTrack imaging and human leukocyte antigen staining confirmed successful engraftment of hMDCs at the injection site. A total of 500,000 passage 3 hMDCs reduced muscle force, whereas later-passage 6 cells had no effect on force production. All treatment groups exhibited small, newly regenerated muscle fibers. In chronic VML injuries, hMDC engraftment using 500,000 passage 6 cells enhanced regenerative characteristics despite limited fusion with host muscle fibers. These findings suggest that hMDCs improved regenerative characteristics in a fibrotic VML model but did not restore contractile force. This work supports the therapeutic potential of hMDCs and provides a foundation for future clinical strategies to treat urinary incontinence.

## 1. Introduction

Urinary incontinence (UI) is a pressing global healthcare issue with a reported prevalence in several countries ranging from 5 to 70%. This wide range demonstrates the variability in reporting since many believe that it is a natural part of aging. Furthermore, there are large differences in the definitions and qualifications of urinary incontinence that associate with variations in its severity amongst males and females [1]. The condition disrupts daily life, leading to social isolation, depression, and a decline in overall well-being [2]. The etiology of UI is multifaceted, with several contributing factors that can stem from injuries to the urethral sphincter, a striated voluntary skeletal muscle that restricts urine flow. These factors can be broadly categorized into myogenic, neurogenic, connective tissue, and hormonal influences [3].

Muscle plays a critical role in urinary incontinence. The rhabdosphincter, a specialized external sphincter composed of skeletal muscle surrounding the urethra, maintains continence during increased intra-abdominal pressure utilizing a voluntary urethral closure mechanism. This striated muscle is composed of both type I (slow) and type II (fast) muscle fibers. These urethral muscles are controlled by three sets of peripheral nerves: sacral parasympathetic (pelvic n.), thoracolumbar sympathetic (hypogastric n.), and sacral somatic nerves (pudendal n.). While the pelvic and hypogastric nerves regulate smooth muscle contraction and relaxation, the pudendal n. controls the external sphincter. Nerve tracts from the central nervous system also terminate at Onuf’s nucleus in the sacral spinal cord and synapse with the pudendal nerves. This provides two pathways to activate urethral closure to restrict urine flow at the external urethral sphincter. The first is via Onuf’s nucleus during sneezing and coughing. The second is the bladder–urethral spinal reflex during laughing, exercise, and lifting heavy objects. Injuries to the striated muscle or innervating nerves are closely tied to UI.

Women are disproportionately affected by UI, accounting for a significant percentage of diagnosed cases. In women, UI often stems from childbirth complications, leading to both muscular and neural deficiencies that disrupt continence mechanisms [4]. Injury severity due to childbirth can result in overstretching, muscle rupture, and/or nerve rupture, resulting in a volumetric loss of skeletal muscle fibers, followed by the development of non-contractile fibrotic tissue [5].

Non-invasive at-home treatments for UI include pelvic floor exercises and behavioral modification [6] whereas other non-invasive approved treatments include occlusive and intravaginal supportive devices. However, these devices often cause patient discomfort, are expensive, and can result in urinary tract infections [6]. Drug treatments for UI include antimuscarinic drugs, α-adrenoreceptor antagonists, β-adrenoreceptor agonists, and antidepressants [7]. While many pharmacological treatments are available, behavioral therapy, solely or in combination with other interventions, is generally more effective than pharmacologic therapies alone in treating both stress and urgency UI [8]. Invasive treatment options include surgical interventions such as transvaginal tension-free transobturator tape (TVT-O) and retropubic tension-free vaginal tape (RTV) [9]. However, these procedures rely on alloplastic materials–synthetic implants placed within the body. The Food and Drug Administration (FDA) has recently issued warnings regarding the use of these materials due to concerns about potential long-term complications, including mesh erosion, infection, and chronic pain [10]. These limitations underscore the urgent need for alternative treatments.

Regenerative medicine offers an alternative approach by using the patient’s own cells to deliver safe and less invasive treatment to the affected area. This autologous cell therapy uses skeletal muscle-derived cells (MDCs) from the patient, injected directly into the rhabdosphincter with the aim of increasing muscle volume and enhancing the sphincter’s ability to resist leakage [11]. Currently, specific challenges hinder clinical implementation of this process. These challenges include muscle damage, atrophy, and fibrosis. Fibrosis poses significant limitations to successfully implement treatment options such as MDC delivery and warrants exploration to determine what challenges need to be overcome.

Optimizing MDC delivery to improve success rates is a crucial step. Present methods do not fully address the complex environment within the damaged sphincter, and optimizing MDC delivery into this type of environment will allow us to gain a better understanding for its effectiveness, advancing UI treatment. This study aims to investigate the engraftment of human MDCs into a controlled pre-clinical injury that mimics the fibrotic environment of the tibialis anterior muscle using volumetric muscle loss (VML). While this muscle is not analogous to the external urethral sphincter, it provides a platform that can be used to study how changes in the MDC injection parameters will affect a fibrotic wound environment. Our hypothesis was that hMDC delivery into fibrotic skeletal muscle wounds would modulate the microenvironment and result in an increase in regeneration. Creating this model in a rodent is challenging. Thus, we used an established VML injury model in the tibialis anterior muscle to mimic a fibrotic muscle injury. While no two muscles are alike, we aimed to gain information about hMDC engraftment in a fibrotic environment to help inform future therapeutic approaches. 

## 2. Materials and Methods

### 2.1. Desmin Quantification

Human muscle-derived cells (MDCs) were obtained from abdominal biopsies at Cook MyoSite (Pittsburgh, PA, USA). MDCs were assessed for use in subsequent experiments using flow cytometry. A total of 100,000 MDCs were blocked with 500 μL of stain buffer (BD 554657), spun down, and the supernatant was aspirated. Cells were fixed with 100 μL Cytofix/Cytoperm (BD 554722) for 20 min, and then 900 μL of perm/wash (BD 554723) was added. Cells were spun and resuspended in 50 μL of 1:200 Isotype control (BD 555746) or 1:100 anti-desmin (Dako M0760) antibody and allowed to stain for 20 min. After 20 min, the solution is diluted in 950 μL of perm/wash and spun. The pellet is suspended in 50 μL of a 1:250 dilution of anti-mouse FITC antibody (Sigma-Aldrich, Burlington, MA, USA, F2883) for 20 min. After 20 min, the solution is diluted in 450 μL of perm/wash and read on a Millipore Guava EasyCyte flow cytometer. The isotype control is used to create a minimum histogram threshold, and then any remainder positive is subtracted from the desmin-stained desmin-positive population.

### 2.2. In Vitro Myogenic Differentiation

A total of 50,000 cells were placed into a 12-well plate with cell growth medium and allowed to grow at 37 °C for 8 days. Upon completion of the incubation, the cells were fixed/permeabilized with ice-cold methanol and then blocked for 15 min with horse serum (Sigma-Aldrich, Burlington, MA, H0146). An amount of 1 mL of 1:250 anti-skeletal myosin (Sigma-Aldrich, Burlington, MA, M4276) is then placed in the well for 45 min in PBS. The well is washed with PBS and stained with 1:250 anti-mouse Cy3 (Sigma-Aldrich, Burlington, MA, C-2181) and DAPI (Sigma-Aldrich, Burlington, MA, D9542). Upon completion, the well is washed and imaged using an Olympus IX83 inverted fluorescence microscope. Two random images taken with a 4x objective within the well are captured and then quantified. An ImageJ (version 1.54) script quantifies the percentage area covered by myotubes and the fusion index (the area fraction of nuclei within myotubes over the total nuclear area).

### 2.3. In Vitro Myogenic Biomarker Analysis

A total of 10,000 cells were seeded into a 48-well plate and grown overnight at 37 °C. Cells were washed with PBS, fixed with 1% Paraformaldehyde for 20 min, washed with PBS and then permeabilized with 0.3% Tx100 for 20 min. After being washed with PBS the plate was blocked with 0.5 mL of 5% Donkey serum (Abcam, Waltham, MA, USA, ab7475) in 0.1% TX-100 for 1 h. Upon completion, the wells were stained with 1:500 Rabbit anti-PAX7 (Proteintech, Rosemont, IL, USA, 20570-1-AP), 1:100 Goat anti-MYOD (Santa Cruz, Santa Cruz, CA, USA, sc-31940), and 1:200 Mouse anti-MYOG (BD, Franklin Lakes, NJ, USA, 556358) for 1 h. The wells were then washed with a blocking agent and stained with 1:250 anti-rabbit:AF+555 (ThermoFisher, Waltham, MA, USA, A32794), 1:250 anti-mouse:FITC (Sigma-Aldrich, Burlington, MA, F2883), and 1:250 anti-goat:Cy5 (Abcam, Waltham, MA, AB6566500UG) for 1 h. Upon completion, the well was washed and imaged using an Olympus IX83 inverted fluorescence microscope (Olympus, Center Valley, PA, USA). Expression was quantified in a combination of manual and automatic counts using the CellSense count and measure software.

### 2.4. Rodent Work

All surgeries were performed humanely under a protocol approved by the Institutional Animal Care and Use Committee at Virginia Commonwealth University (Protocol# AD10000675, 3/27/2025) and followed the National Institutes of Health guide for the care and use of laboratory animals. Each animal was individually housed in a ventilated polysulfone cage in an ALLAC accredited animal facility with temperature and humidity regulation appropriate to the species with a 12 h/12 h light/dark cycle. Food pellets and water were provided ad libitum, and animals were acclimated to the new housing a week prior to surgery.

### 2.5. In Vivo Cell Injection Validation

Rats were injected with cardiotoxin in the right tibialis anterior to induce muscle injury (see injury model below). After 24 h, cells were prepared for injection. Passage three myoblasts were used as the test for this injection model. This is because higher-passage MDCs did not retain the dye as efficiently in validation studies. Passage three myoblasts were thawed and centrifuged at 1000× *g* for 5 min, then resuspended in 2.0 mL of warm, 1x PBS. The cell suspension was added to 2.0 mL VivoTrack 680 in sterile, warm, 1x PBS and incubated in the dark for 15 min. The solution was diluted with 15.0 mL 1x PBS and spun down at 1000× *g* for 5 min, for a total of three washes. The final suspension was 1.39 × 10^7^ cells/mL in 200 µL 1x PBS. A 300 µL insulin syringe was used for three injections of 50 µL of cell suspension into the tibialis anterior 1 day post-injury. Myoblast engraftment was observed using an IVIS Spectrum Preclinical In Vivo Imaging System. Bioluminescent signal from each transplanted animal was analyzed to determine the level of engraftment.

### 2.6. Cardiotoxin Animal Model

Immunocompromised female RNU rats weighing 200–250 g (Athymic nude rats, Hsd: RH-*Foxn1^rnu^*, Charles River Laboratories, Worcester, MA, USA) were used for all experiments (*n* = 8 animals/group). The left lower limbs were shaved and injection sites marked. A muscle injury was created in the left tibialis anterior muscle using 150 µL of 10 mM cardiotoxin. The right lower limbs were left untreated. Twenty-four hours after cardiotoxin injection, treatment limbs were injected with PBS, 100,000 passage 3 (p3) cells (100P3), 500,000 p3 cells (500P3), or 500,000 passage 6 (p6) cells (500P6). Tibialis anterior muscles were harvested at 7 days post-injury. Animals were chosen at random.

### 2.7. Volumetric Muscle Loss Animal Model

Animals were anesthetized using aerosolized 4–5% isoflurane/400 mL/min O_2_ until unconscious, and anesthetization was maintained during preparation and surgery with 2.5% isoflurane, 400 mL/min O_2_. The hindlimb surgical site was shaved and cleaned with 3x alternating swabs of isopropanol and chlorhexidine. Eight female RNU rats were used for all experiments and animals were randomly selected for the surgical procedure. The right lower limbs were shaved, and incisions were made to expose the tibialis anterior muscle. A full-thickness 5 mm biopsy punch was made in the tibialis anterior muscle. Nylon sutures were placed to mark the injury margins as a way to identify the injury site for the remaining treatments and analysis. The left hindlimbs acted as untreated controls. The skin of the injured limb was closed, and the wounds healed for 6 weeks to mimic a fibrotic environment. The animals were then injected with either 500,000 p6 human MDCs derived from a vastus lateralis muscle or PBS to treat the injuries at 6 weeks. Passage six MDCs were chosen for the VML model, as this is the passage used in current clinical trials. Tibialis anterior muscles were assessed at 7, 8, and 10 weeks post-injury (*n* = 8 per time period).

### 2.8. Muscle Force Testing

Muscle force measurements were performed in vivo using the 1300A Whole Animal System (Aurora Scientific, Aurora, ON, Canada). Animals were anesthetized using aerosolized 4–5% isoflurane/400 mL/min O_2_. Observers were blinded prior to anesthesia. Following anesthesia, each rat was placed in a supine position on the sterile device platform. Each leg was individually fixed at the knee with the paw pressed firmly onto the footpad. Two electrodes were inserted in the subdermal space of the tibialis anterior muscle with the positive electrode proximal to the negative electrode. Stimulating voltages and signal frequency were optimized for each limb before recording data. Twitch stimulations were performed first with <1 ms electrical pulses and a ~1 s rest period between pulses. Tetany measurements were performed with 500 ms stimulation and a 120 s resting period. Total times, maximum contraction rate, minimum contraction rate, maximum force, and minimum force were recorded for each test. Total time was defined as the total time for a single-twitch or tetanic contraction (including time to peak contraction and time to full relaxation). Contraction rate was defined as the force generated per unit time. Maximum force was defined as the peak force generated, while minimum force was defined as the lowest force value within the data set.

### 2.9. Gene Expression Analysis

To isolate total RNA, a 30–50 mg sample was taken from the injury site, minced, and transferred to a Bead Bug Zirconium 6 mm Bulk Bead tube (Benchmark Scientific, Sayreville, NJ, USA) containing 1 mL TRIzol (ThermoFisher Scientific, Waltham, MA, USA). Tubes were processed for 60 s at max speed three times through the device with a 10 min rest period on ice between runs. Isolated RNA concentration and purity were measured after isolation using a spectrophotometer at 260 nm and 280 nm wavelengths (BioTek Take3, BioTek Synergy H1, Winooski, VT, USA). cDNA was generated from the total RNA with a High-Capacity cDNA Reverse Transcription kit (Life Technologies, Carlsbad, CA, USA) according to the manufacturer’s instructions using the supplied random primers. Quantitative PCR was performed with Power Sybr Green (Fisher Scientific, Hampton, NH, USA) with optimized sample dilutions and melt curve analysis using custom designed primers (Fisher Scientific) and pre-validated primers (Bio-Rad Hercules, CA, USA). *Paired box 7 (Pax7), myogenic factor 5 (Myf5), myogenin (Myog), myogenic factor 6 (Myf6), myosin heavy chain 1 (Myh1), myosin heavy chain 7 (Myh7), platelet-derived growth factor receptor alpha (Pdgfrα), connective tissue growth factor (Ctgf), apolipoprotein E (Apoe), intercellular adhesion molecule 1 (Icam1), integrin alpha 7 (Itgα7),* and *monocyte chemotactic protein 1 (Mcp1)* were quantified using standard curves created by known dilutions of muscle lysates normalized to *Gapdh* (Table 1). Data were represented as treatment/control. To calculate treatment/control, 2^−ΔΔCt^ values were determined for each treatment group and their respective contralateral control group (normalized to *Gapdh*). Values for contralateral control groups were used to normalize expression values for the respective treatment groups. *Gapdh* levels were consistent across treatment groups.

### 2.10. Tissue Measurements and Histology

Tibialis anterior muscle was harvested, placed on a blue drape beside a ruler, and imaged using a Nikon T3 Rebel Camera with stand to maintain camera height. Muscle width and length were measured using Fiji 2.14.0. Muscles were then prepared in 4% paraformaldehyde (VWR, Radnor, PA, USA), dehydrated using a series of ethanol and xylene washes, embedded in paraffin wax, and sectioned at 5 µm thickness. The sections were placed on Histobond slides (VWR), deparaffinized [12]. Slides were stained with hematoxylin (Sigma-Aldrich, St. Louis, MO, USA) and eosin (Sigma-Aldrich). Coverslips were mounted with xylene-based mounting media then dried in a chemical hood. Images were taken at 10× magnification with the Axio Observer.Z1 (Zeiss, Jena, Germany). Regions of interest were selected to mark non-injured and injured areas. For each tissue sample, three non-injured and three injured regions were selected for the analysis and quantification of Feret diameter (a measure of fiber size) and centrally located nuclei (a marker of regenerating fibers). Three independent, blinded observers analyzed each image.

### 2.11. Immunohistochemistry

The OPAL staining platform was used for multiplex IHC analysis of muscle tissue sections using the Opal 4-color anti-rabbit automation IHC kit (NEL830001KT, Akoya Biosciences, Marlborough, MA, USA) as we have shown previously [13]. Slides were prepared following the Opal Multiple IHC assay development guide with primary target detection performed for MyHC-I (Opal 690), Pax7 (Opal 480), laminin (Opal 620), MyHC-II (Opal 570), human leukocyte antigen (HLA) (Opal 520), and counter-stained with DAPI (Table 2). Epitope retrieval was performed with heat induced BOND ER solution-1 citrate pH 6 (AR9961, Leica Biosystems, Wetzlar, Germany) or BOND ER solution-2 EDTA pH9 (AR9640, Leica Biosystems) incubating the slides in solution at 95 °C for 20 min. Slides were blocked with PKI blocking buffer (ARD1001EA, Akoya Biosciences). Both primary and secondary antibodies were diluted in 1x plus automation amplification solution (FP1609, Akoya Biosciences). Opal fluorophore signals were amplified by incubating in each wavelength’s respective Opal signal reagent (1:150 dilution) in 1x plus automation amplification solution. Coverslips were mounted with ProLong™ diamond antifade solution (P36970, Invitrogen, Carlsbad, CA, USA). All morphometric analyses were performed with blinded reviewers. Morphometrics were quantified for laminin and HLA by counting the total number of positive pixels from the total ROI pixels taken as a percentage. Pax7 positive cells were counted as those that had overlapping nuclear stain and Pax7 stain per ROI. MyHC-I and MyHC-II positive fibers were counted per ROI.

### 2.12. Statistical Analysis

All statistical analyses were performed using GraphPad Prism (11.1.0). The animal number was chosen based on a power analysis using an alpha of 0.05 and a power of 80% (delta = 5, sigma = 3, m = 1) to reveal a minimum of *n* = 7 per group to yield statistical significance. Data are presented as mean ± standard error of the mean (SEM) unless otherwise stated. The specific statistical tests used were chosen based on the distribution of the data and the number of groups compared. For comparisons between two groups, a two-tailed Student’s *t*-test was used. For comparisons involving more than two groups, a one-way analysis of variance (ANOVA) was used, followed by Tukey’s post hoc test to determine significant differences between individual groups. Muscle force testing data was analyzed using two-way ANOVA to account for the within-subject comparisons of contralateral and treated limbs across different time points, followed by Tukey’s post hoc test. Gene expression data, histological measurements (Feret diameter, centrally located nuclei), and immunohistochemistry quantification were analyzed using ANOVA followed by Tukey’s post hoc test. Groups that share a letter are not statistically significant from one another, and groups that do not share a letter are statistically significant from one another by a *p*-value of at least 0.05.

## 3. Results

### 3.1. Muscle Force Testing Following Cardiotoxin Injury

**Figure 1:**
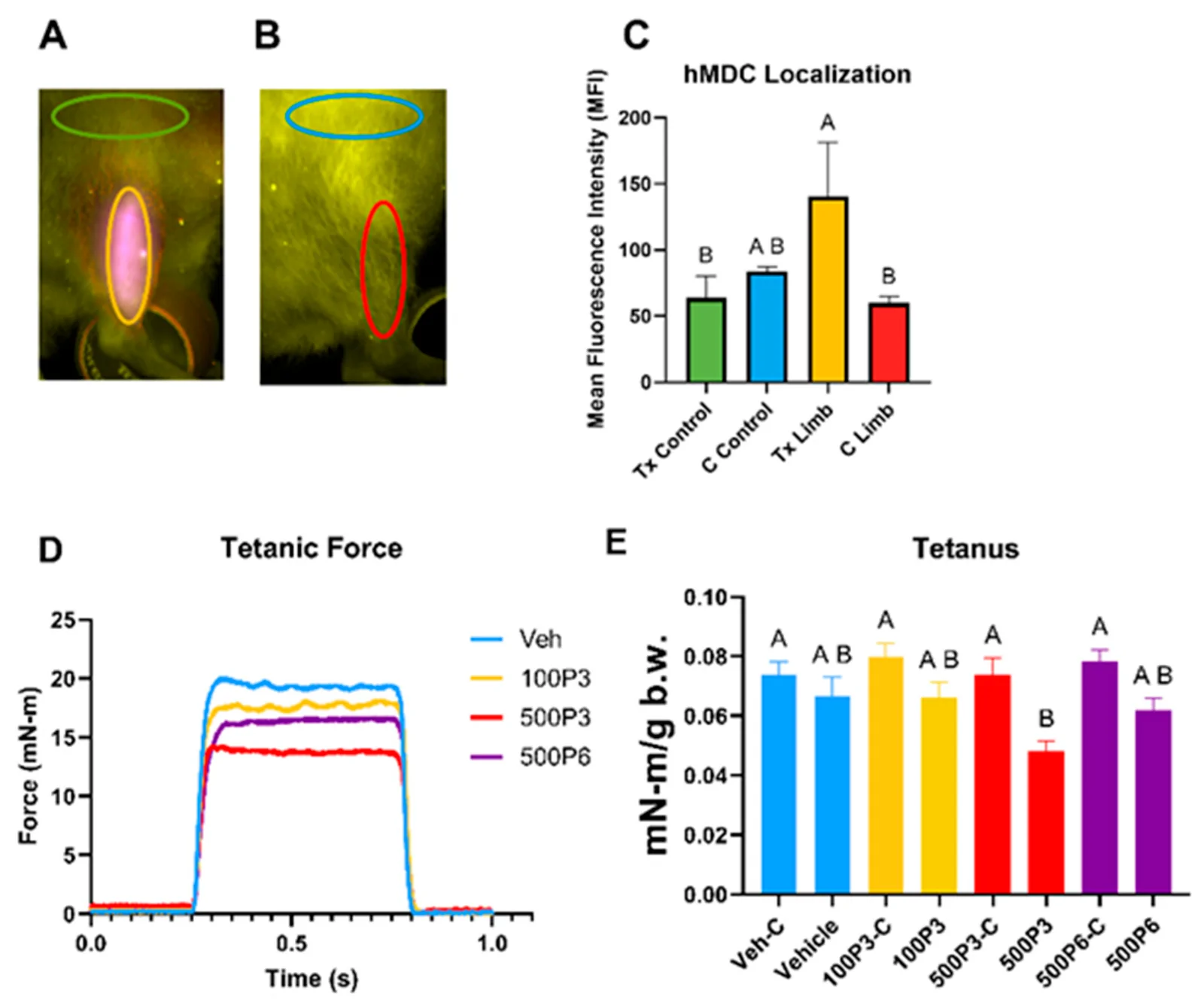
Muscle force following hMDC injection. hMDCs were tagged with a cellular dye and injected into cardiotoxin injured muscle (**A**,**B**). The limb was imaged at 660 and 720 nm and intensity quantified, demonstrating localization at the injection site. Green and blue circles denote control background for subtraction, while yellow and red circles denote measured injection area (**C**). Tetanic muscle force was measured demonstrating reduced force only in the 500P3 treatment group (**D**,**E**). Groups not sharing a letter are statistically significant with α = 0.05. Data shown are means ± SEM of 8 animals (N = 8).

Prior to muscle injury injections, hMDCs were characterized for desmin levels (Supplemental Figure S1A), percentage of the surface area covering the plate during differentiation (Supplemental Figure S1B), and fusion index (Supplemental Figure S1C). p3 MDCs covered more of the plate and formed more myotubes compared to p6 MDCs; however, these results were only qualitative due to lower n-values. hMDCs were stained using VivoTrack dye, and dye intensity was measured using IVIS imaging. Results showed an increased level in hMDC-treated legs (*p* = 0.0175) versus the PBS-injected control (Figure 1A–C). Muscle force testing was conducted 7 days post-cardiotoxin injury. Interestingly, absolute force output was highest in vehicle control limbs and was lowest in the 500P3 hMDC-treated limbs (Figure 1D). These results were corroborated when absolute force was normalized to body weight (g), where the 500P3 group was significantly lower (*p* = 0.0081) compared to the contralateral limb, indicating a protracted recovery period for force restoration. All other treatment groups did not differ significantly from their respective contralateral controls (Figure 1E). Time to maximum rate of contraction and rate of relaxation were measured (Supplemental Figure S2A,B) and demonstrated no differences in the rate of contraction but did show an increased rate of relaxation in 100P3 treatment groups compared to the contralateral control leg (*p* = 0.0127).

### 3.2. Gene Expression Following hMDC Treatment in Cardiotoxin Injuries

**Figure 2:**
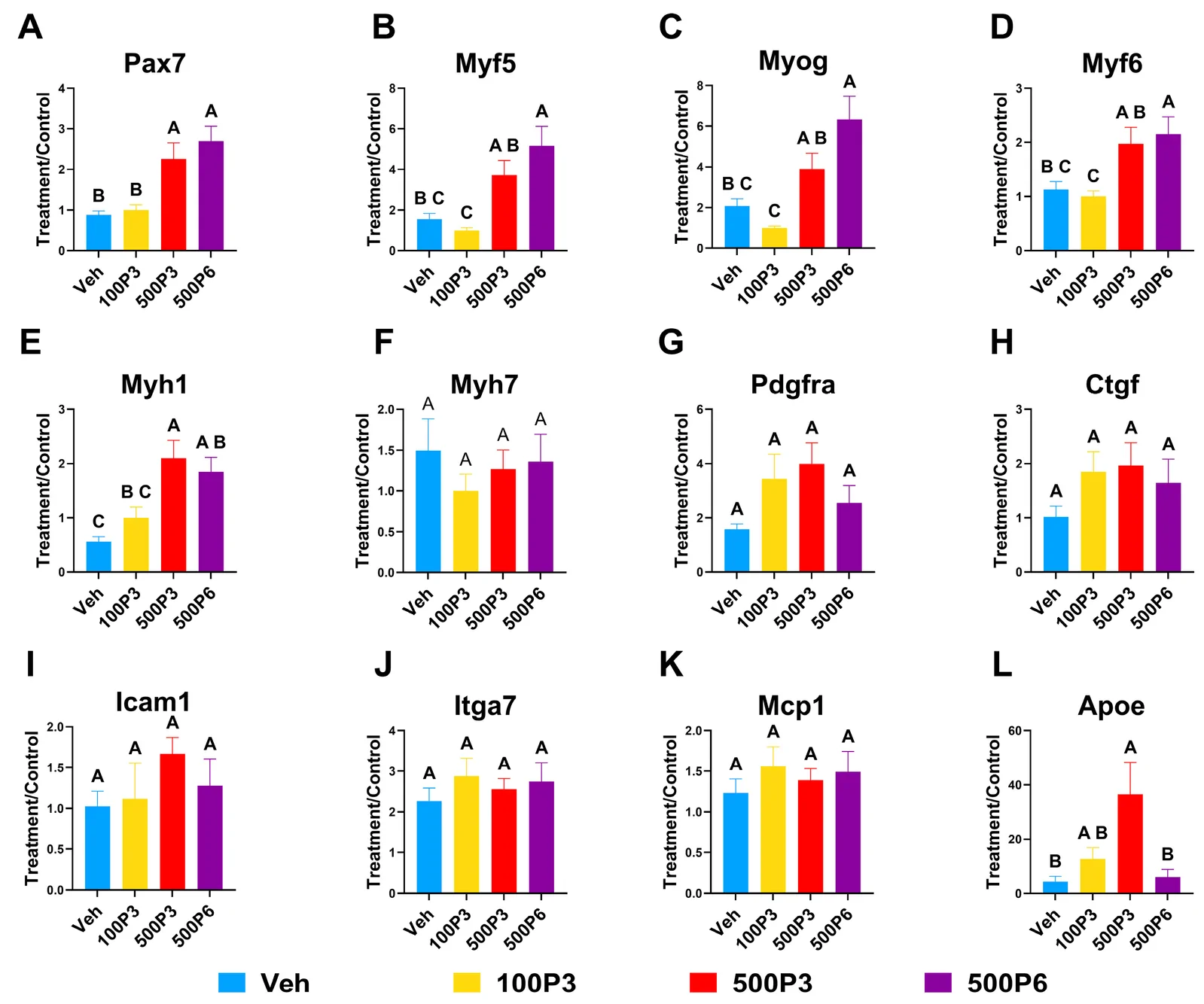
Myogenic-associated genes increased in hMDC-treated limbs. Pax7, Myf5, Myog, and Myf6 showed elevated myogenic gene levels in animals treated with a higher cell dose (**A**–**D**). Myh1 mRNA levels were highest in 500P3-treated limbs and 500P6 was similar to 500P3 (**E**). Myh7, Pdgfra, Ctgf, Icam1, Itga7, and Mcp1 were all unchanged by cell treatments (**F**–**K**). Apoe was highest in 500P3-treated limbs (**L**). Groups not sharing a letter are statistically significant with α = 0.05. Data shown are means ± SEM of 8 animals (N = 8).

Tissue samples were harvested 7 days post-injury, immediately following muscle force testing. A portion of each sample was used for gene expression analysis (Figure 2). The expression of myogenic and cell engraftment-related markers were examined to assess the effects of myoblast injection on muscle repair and recovery. Quantitative analysis revealed a significant increase in mRNA for early myogenic regulatory factors (MRFs), including Pax7 (*p* = 0.0137), Myf5 (*p* = 0.0003), Myog (*p* = 0.0039), and Myf6 (*p* = 0.0336), in muscles treated with 500k cells regardless of passage number when compared to the vehicle control. Pax7, a marker of skeletal muscle stem cells (MuSCs), significantly increased in the 500P3 (*p* = 0.0137) and 500P6 (*p* = 0.0009) groups compared to the vehicle control, whereas the 100P3 group did not differ significantly from the vehicle (Figure 2A). Myf5, an early-stage myogenic marker indicating commitment of MuSCs to the myogenic program, was also upregulated in the 500P3 versus 100P3 (*p* = 0.0216) and 500P6 versus 100P3 (*p* = 0.0003) and vehicle (*p* = 0.0016), suggesting enhanced myogenic commitment and differentiation with higher cell numbers (Figure 2A–D). Myog, a marker of the fusogenic phase of myogenic differentiation, was elevated in 500P3 versus 100P3 (*p* = 0.0431) and 500P6 versus vehicle (*p* = 0.0039) and 100P3 (*p* = 0.0001) treatments. Myf6, a late-stage marker of myogenic differentiation, was higher in the 500P3 (*p* = 0.0243) and 500P6 (*p* = 0.0336) groups compared to the 100P3 group, while the vehicle control was statistically similar to both 100P3 and 500P3 treatments. Myh1, a marker of fast-twitch fibers, showed increased expression in the higher cell number groups compared to the vehicle control (*p* = 0.0006 and *p* = 0.0041) (Figure 2E). In contrast, Myh7, a marker of slow-twitch fibers, showed similar expression across all groups (Figure 2F), indicating no effect of the treatments on slow-twitch fiber development. There were no significant differences in the expression of Pdgfrα, Ctgf, Icam1, Itgα7, or Mcp-1 (Figure 2G–I). Apoe, a cholesterol regulator, increased in the 500P3 group compared to the vehicle control (*p* = 0.0103) and to 500P6 (*p* = 0.0207), while the 100P3, 500P6 and vehicle showed similar expression levels.

mRNA expression were compared between vehicle and control legs using 2^−ΔΔCt^ values. These values were similar for almost all myogenic markers (Supplemental Figure S3). Myog (Supplemental Figure S3B) and Pdgrfa (Supplemental Figure S3G) expression was higher in the vehicles compared to the contralateral limbs. We also reported mRNA expression levels using 2^−ΔΔCt^ values to compare all treatment groups together. Contralateral limbs were represented using two dashed lines to display the variance within the sample. Pax7 expression was elevated in the 100P3 and 500P3 treatment groups compared to the vehicle (*p* = 0.0339 and *p* = 0.0035), and 500P6 was similar to both the P3 treatment group and the vehicle (Supplemental Figure S4A). Myf5 (*p* = 0.0085), Myog (*p* = 0.0282), and Myf6 (*p* = 0.0268) expression were highest in 500P3 treatments, and P3 treatments were consistently elevated above the controls (Supplemental Figure S4B–D). Myh1 was elevated in all cell-treated groups (*p* = 0.0003, *p* = 0.0024, and *p* = 0.0098) (Supplemental Figure S4E), while Myh7, Pdfra, Ctgf, Icam1, Itga7, and Mcp1 were all similar (Supplemental Figure S4F–K). Apoe was highest in 500P3 hMDC treatments (*p* = 0.0134), 100P3 and 500P6 were similar, and the vehicle was similar to the contralateral limb (Supplemental Figure S4L).

### 3.3. Morphometric Analysis of H&E-Stained Tissue Sections in Cardiotoxin Injuries

**Figure 3:**
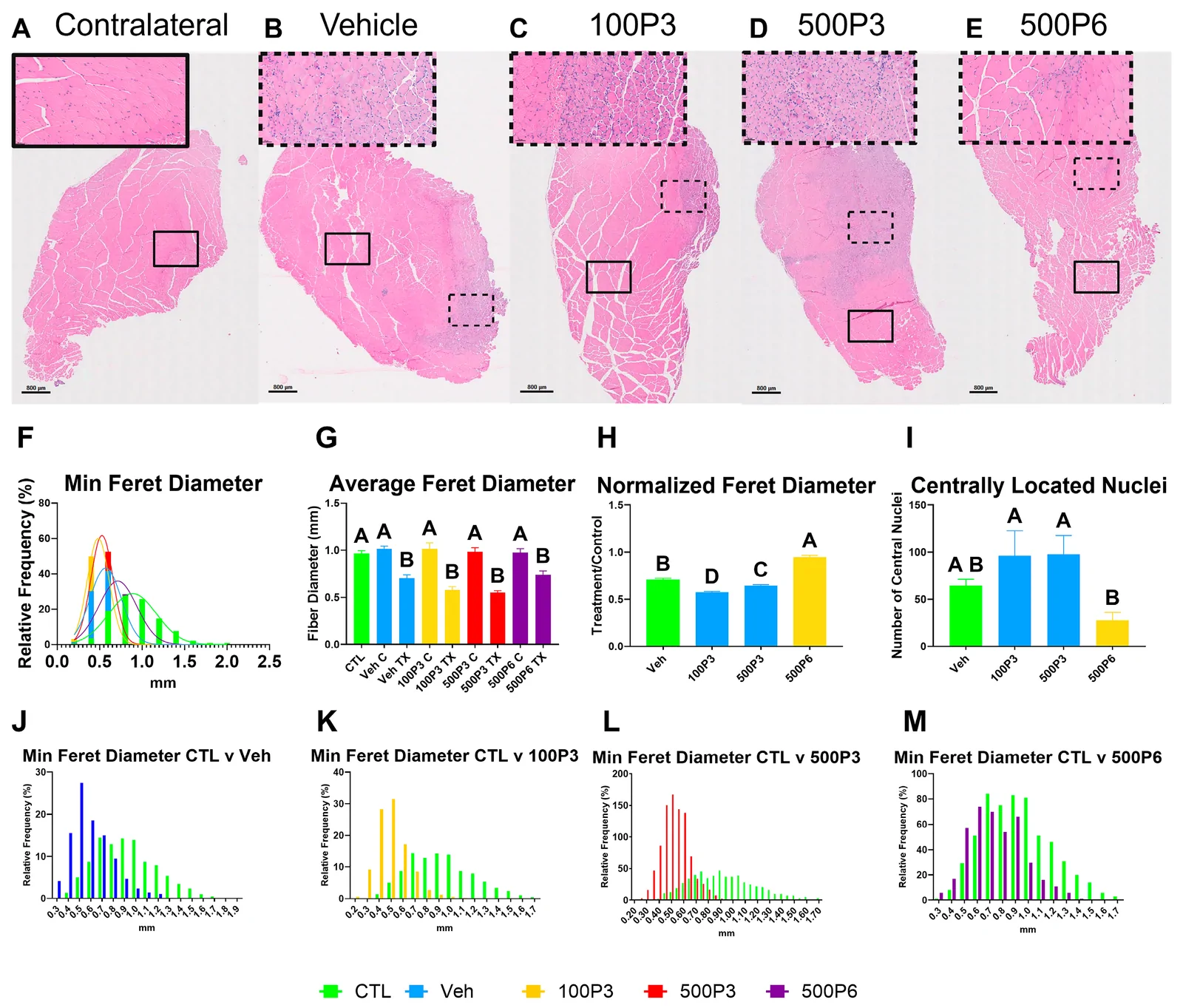
hMDC treatments increase the number of small-diameter fibers. Minimum Feret diameter was measured using hematoxylin and eosin-stained sections. Representative sections are shown in (**A**–**E**). Solid boxes indicate control ROIs within the same tissue section, while dotted lines represent injury ROIs with or without treatments (Tx). Minimum Feret diameter measurements demonstrated smaller fibers in cell-treated groups (**F**). Average minimum Feret diameter measurements were compared between internal tissue controls (solid line) and injury/treatment sites (dotted line) (**G**). Feret diameter of treatment groups was then normalized to their internal tissue controls (**H**). Centrally located nuclei (CLN) were counted, demonstrating more CLN in P3-treated injuries (**I**). Relative frequency histograms show the shift in fiber diameter when each group is compared to the contralateral control (**J**–**M**). Groups not sharing a letter are statistically significant with α = 0.05. Data shown are means ± SEM of 8 animals (N = 8).

Quantitative analysis of histology demonstrated a significant reduction in muscle fiber diameter in muscles treated with 500P3 cells. Figure 3A–E show representative images from each group stained with H&E. Solid-line boxes indicate regions of interest (ROIs) in non-injured tissue, while dashed-line boxes depict ROIs in the injured site. The 500P3 group exhibited the most significant reduction in fiber diameter in the injured region, whereas the 500P6 group showed fiber diameters similar to those of the contralateral, uninjured muscle. Furthermore, the number of regenerating fibers was lower in the treated groups compared to healthy, fully formed fibers. Overall, the data showed that cell treatment skewed Feret diameter towards smaller values compared to the control (Figure 3F). The contralateral limb in each animal was unaffected by cell treatments in the injured muscle (Figure 3G). Analysis of non-normalized Feret diameter data showed an increase in fiber diameter due to the 500P6 treatment when compared to the contralateral control limb (*p* < 0.0001). This was in contrast to the 500P3 and 100P3 MDC treatments that resulted in smaller fiber diameters (*p* < 0.0001) (Figure 3H). Feret diameter in the treated limbs was next compared to the contralateral limbs, accounting for individual animal variation. Again, smaller fiber diameters in MDC-treated groups persisted and were statistically significant (*p* < 0.0001) compared to the vehicle control (Figure 3H). Group-specific comparisons to contralateral limbs revealed a substantial shift towards smaller fibers in the vehicle, 100P3, and 500P3 groups (Figure 3J–L). In contrast, the 500P6 group showed a Feret diameter distribution nearly identical to its respective contralateral limb (Figure 3M).

### 3.4. Immunohistochemical Analysis in Cardiotoxin Injuries

**Figure 4:**
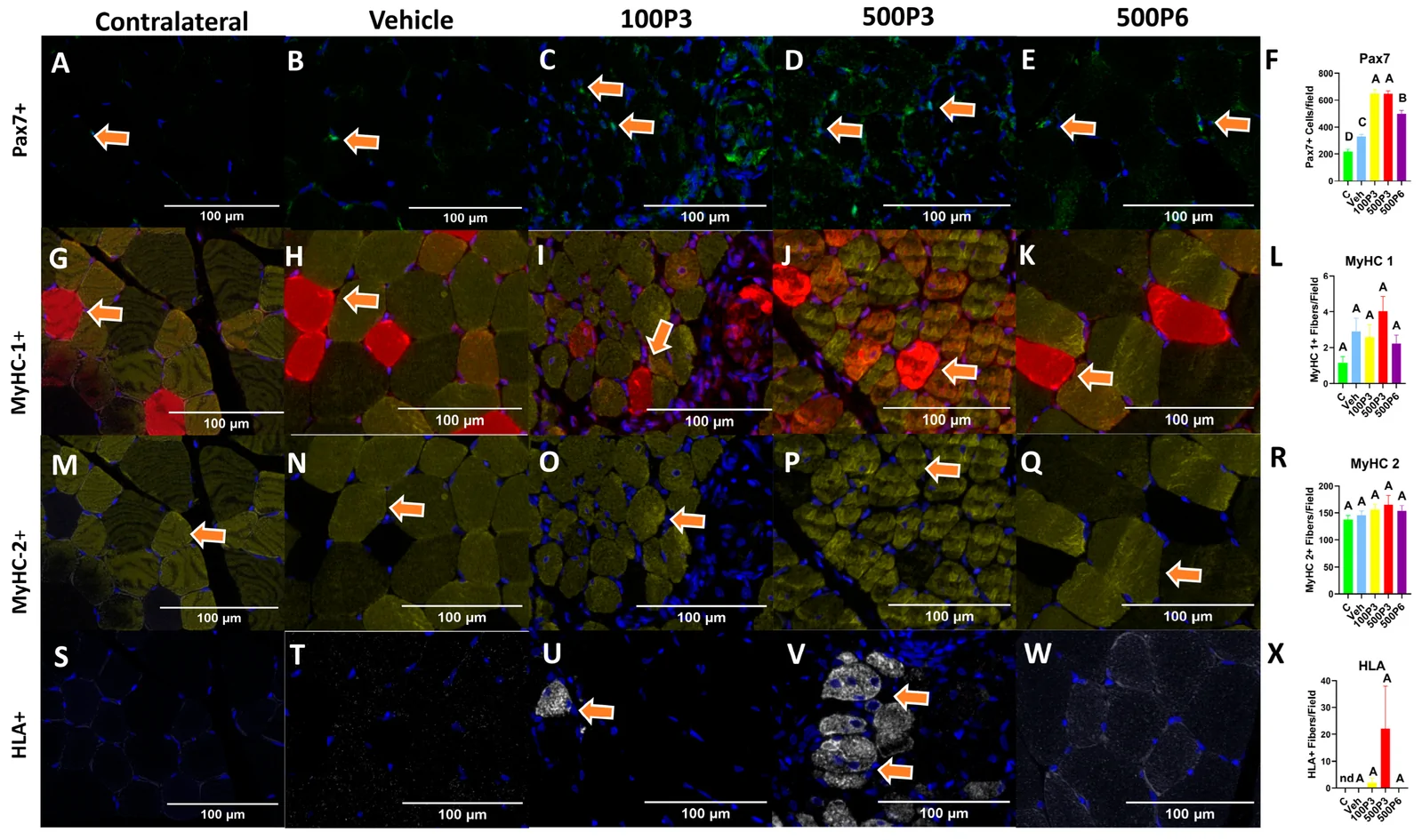
hMDC treatments improve markers of skeletal muscle regeneration. Pax7+ cells were labeled and counted per field, revealing that hMDC treatments increased the number of muscle stem cells compared to vehicles and contralateral control as indicated by orange arrows (**A**–**F**). MyHC-1 (slow) fibers (orange arrows) did not change with treatment (**G**–**L**). MyHC-2 (fast) fibers (orange arrows) were also unchanged by treatments (**M**–**R**). HLA staining showed an increase in the average number of HLA+ fibers (orange arrows), while no staining was detected in either vehicle or contralateral muscle samples (**S**–**X**). Groups not sharing a letter are statistically significant with α = 0.05. Data shown are means ± SEM of 8 animals (N = 8).

Representative immunohistochemistry images for Pax7, MyHC-1, and MyHC-2 were selected from each experimental group and quantification detailed at the end of each data row. Pax7-positive cells (green), representing muscle stem cells located at the periphery of muscle fibers, were quantified. The contralateral, untreated (C) group exhibited the lowest number of Pax7+ cells per sample, while the vehicle group demonstrated a marginally higher count. hMDC treatments at p3 showed statistically significant increases (*p* < 0.0001) in Pax7+ cell counts compared to all other experimental groups, while the p6 MDC treatment exhibited a significant increase (*p* = 0.0004) compared to the contralateral limb (Figure 4F). MyHC-1-positive cells (red), indicating slow-twitch muscle fibers, showed no significant differences in counts among any groups (Figure 4L). Similarly, MyHC-2-positive cells (yellow), denoting fast-twitch muscle fibers, showed no statistically significant differences between the groups (Figure 4R). Human leukocyte antigen (HLA)-positive cells (white) were examined to assess the engraftment of human MDCs into the host tissue. While there was a numerical increase in HLA-positive cells in the 500P3 group, the high variance between samples prevented this difference from reaching statistical significance (Figure 4U,W). HLA-positive cells were also observed in the 100P3 group, again with high inter-sample variability (Figure 4T,W). Double positive staining for Pax7/HLA and MyHC-1/HLA were measured. It was determined that 500P3-treated muscles showed a significant increase (*p* < 0.05) in Pax7/HLA double positive cells, while 100P3-treated muscles showed some double positive staining, but it was not statistically significant (Supplemental Figure S5A–F). Next, we measured MyHC-1/HLA double positive fibers and demonstrated that engrafted hMDCs were incorporated with type 1 fibers and we did not detect positive HLA staining in type 2 fibers (Supplemental Figure S5I,J). However, the inherent variability within the samples did not demonstrate significance (Supplemental Figure S5L). HLA-positive fibers were observed in 500P6 tissue sections, but intensity levels were much lower compared to 500P3. Further investigation into whether 500P6-treated injuries stained positively for HLA at lower levels showed a significant increase in 500P6-treated limbs compared to contralateral limb (*p* < 0.0001) and vehicle controls (*p* < 0.0001) (Supplemental Figure S6).

### 3.5. Muscle Force Testing Following VML

Gross morphometric analysis of injured muscles was determined, and no apparent differences between PBS and cell-treated muscles were observed (Supplemental Figure S7A–C). Body weights were also not statistically different between PBS- and hMDC-treated animals and those animals continued to gain weight over the 0–10-week period (Supplemental Figure S7D). Muscle length did not show any appreciable differences (Supplemental Figure S7E). Muscle width was similar in contralateral control legs at 7, 8, and 10 weeks, and injured legs were not statistically different from controls at weeks 7 and 8. By week 10, muscle width was significantly lower in PBS-treated animals compared to contralateral control legs (*p* = 0.006), indicating that muscle atrophy occurred by week 10 to reduce muscle size (Supplemental Figure S7F).

**Figure 5:**
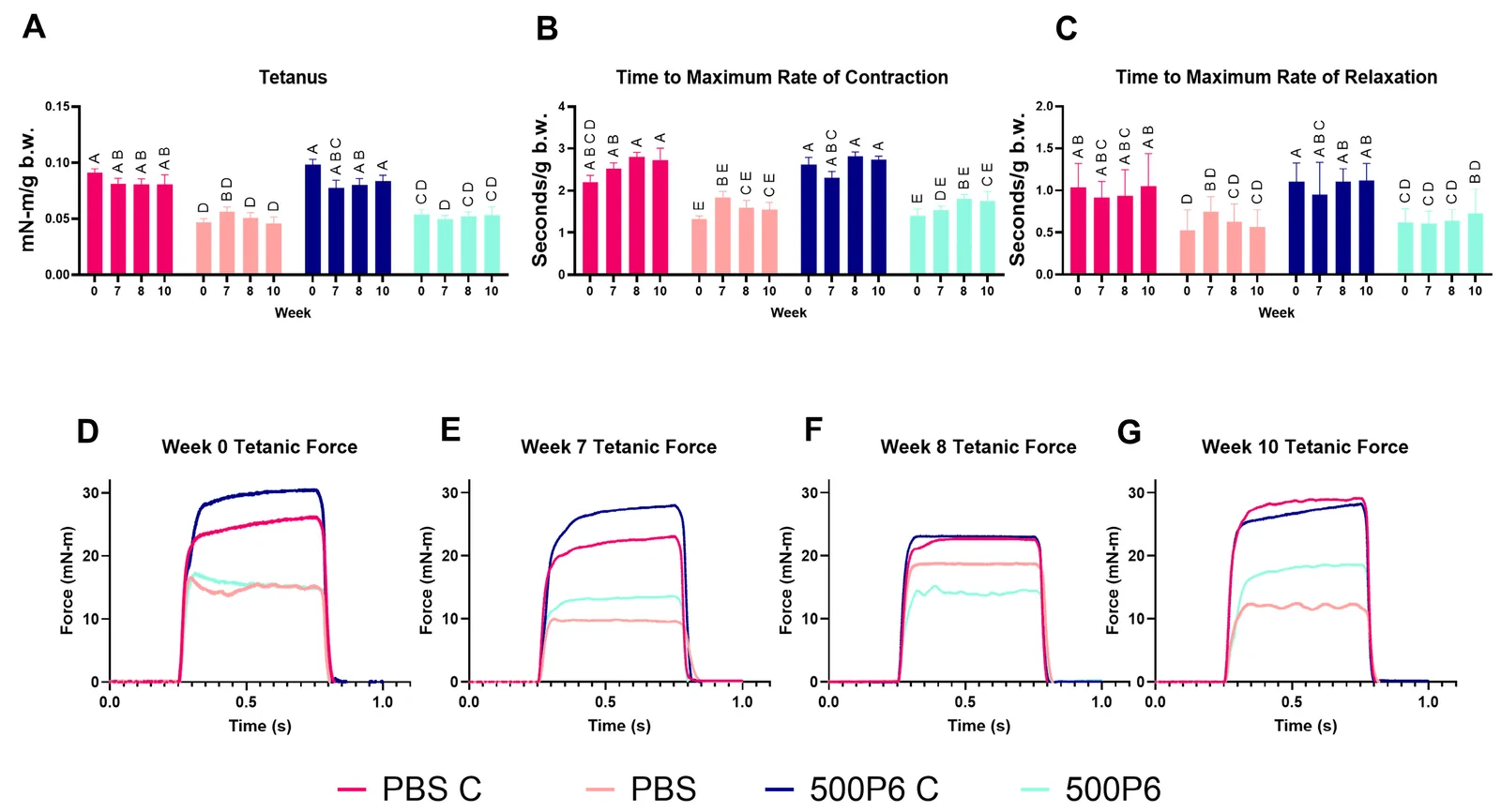
Tetanic muscle force analysis. Muscle force was reduced in PBS and hMDC-treated limbs (**A**). Time to maximum rate of contraction showed a significant reduction, which aligned with the reduced force (**B**). Time to maximum rate of relaxation was significantly lower, also due to reduced force (**C**). Representative tetanic force curves demonstrate the effect of the VML injury on muscle force and the effect of each treatment (**D**–**G**). Groups not sharing a letter are statistically significant with α = 0.05. Data shown are means ± SEM of 8 animals (N = 8).

Muscle force testing was conducted at 0, 7, 8, and 10 weeks post-injury in the longitudinal study. Peak tetanic force was highest in the contralateral controls. Both the PBS-treated and cell-treated groups exhibited significantly lower tetanic force compared to the control groups (*p* = 0.0083 and *p* = 0.028) but did not differ significantly from each other at any time point (Figure 5A). A similar trend was observed for the time to maximum rate of contraction (*p* < 0.0001 and *p* < 0.0001) (Figure 5B) and relaxation (*p* < 0.0001 and *p* < 0.0001) (Figure 5C), with both control groups showing higher values compared to both treatment groups. There were no significant differences in these parameters within each group over time. Time–force curves for tetanic force at each time point (0, 7, 8, and 10 weeks post-injury) are shown in Figure 5D–G. The curves for weeks 0, 7, and 10 showed similar force–time relationships. At week 0, there was a clear VML injury with reduced force production in the injured limb (Figure 5D). At week 7, the injury persists (Figure 5E). At week 8, the injured limb in the PBS-treated animals is closer to control levels, but control levels were lower than expected (Figure 5F). At week 10, cell-treated limbs appear to increase force to a slightly higher degree than PBS-treated muscles, but these data are not statistically significant (Figure 5G).

### 3.6. Gene Expression Analysis: VML

**Figure 6:**
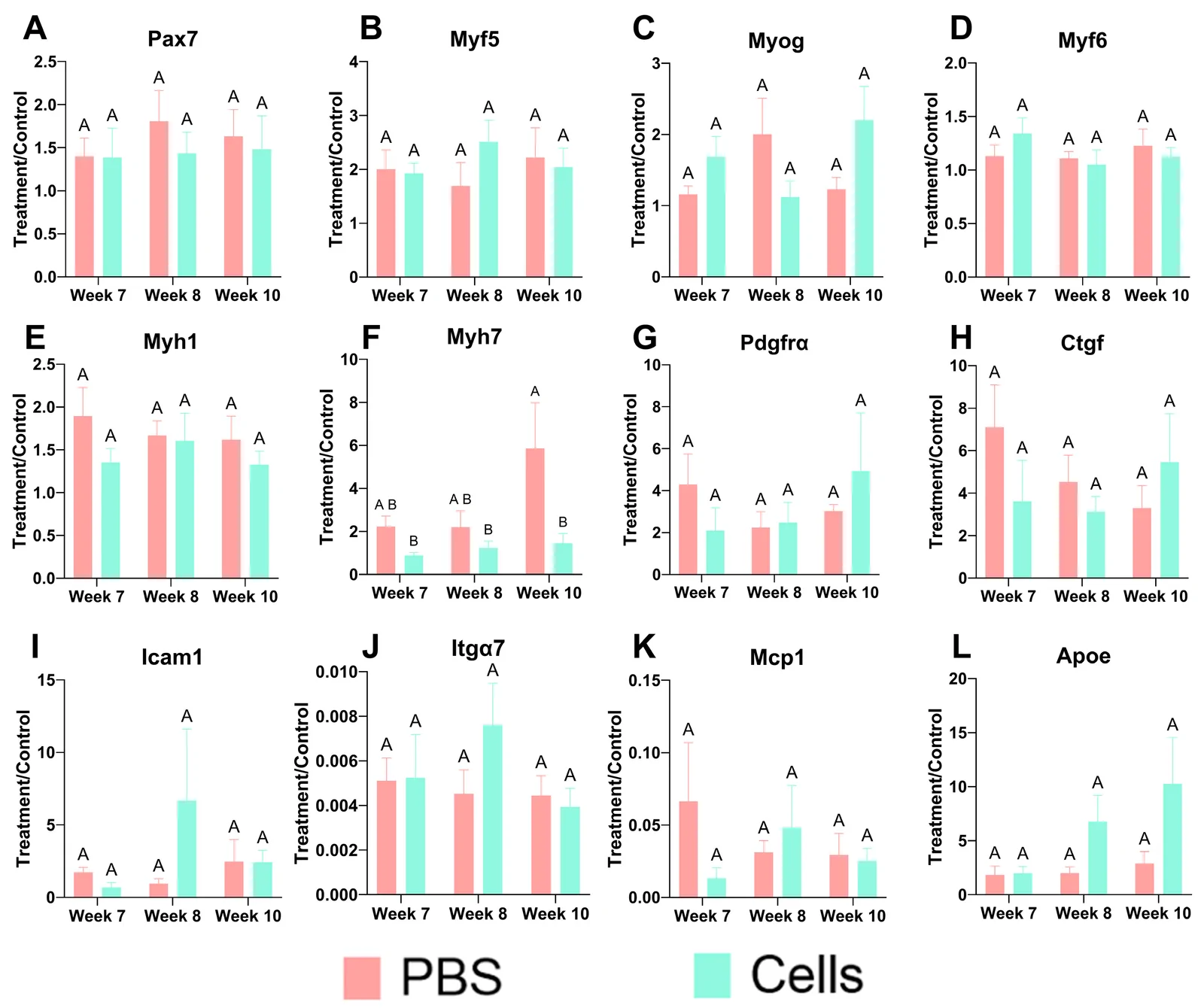
Slow myosin heavy chain mRNA levels increased without cell treatment. Myogenic gene expression was similar for all treatment groups (**A**–**D**). Myh1 fast-twitch gene expression was also similar regardless of treatment (**E**). Myh7 slow-twitch gene expression was elevated by week 10 in PBS-treated injuries, while cell-treated injuries remained unchanged (**F**). Pdgrfa, Ctgf, Icam1, Itga7, Mcp1, and Apoe were all unchanged by PBS or cell treatment (**G**–**L**). Groups not sharing a letter are statistically significant with α = 0.05. Data shown are means ± SEM of 8 animals (N = 8).

Tissue samples from the injury site of the tibialis anterior muscle were harvested at weeks 7, 8, and 10 post-injury. Gene expression of markers for myogenesis, fibrosis, and cell adhesion was analyzed using treatment/control as shown in Figure 2. No significant differences were detected in Pax7, Myf5, Myog, Myf6, and Myh1 expression (Figure 6A–E). Myh7 showed a significant increase at week 10 in PBS-treated legs compared to hMDC-treated legs (*p* = 0.046) (Figure 6F). Pdgrfa, Ctgf, Icam1, Itga7, Mcp1, and Apoe were all similar (Figure 6G–L).

mRNA expression values were also analyzed using 2^−ΔΔCt^. Similarly, there were no significant differences in the expression of the myogenic markers Pax7, Myf5, Myog, or Myf6. Myh1, a marker of fast-twitch muscle fibers, showed no significant differences between groups (Supplemental Figure S8A–E). Myh7, a marker of slow-twitch fibers, showed a slight increase in expression in the PBS group over time (*p* = 0.0116), but remained relatively constant in the cell-treated group (Supplemental Figure S8F). Pdgfra, a marker of fibro-adipogenic progenitor cells (FAPs), showed similar expression levels in both the PBS and cell groups at weeks 7 and 8. However, both groups showed a significant increase in Pdgfra expression at week 10 (*p* < 0.0001 and *p* < 0.0001), potentially indicating a later role for FAPs in the regenerative process or an expansion of the FAP population (Supplemental Figure S8G). Ctgf expression showed a significant increase at week 10 in the PBS group (*p* = 0.0415), while remaining relatively constant across the three weeks in the cell-treated group (Supplemental Figure S8H). The expression of Integrin α7, Mcp1, Apoe, and Icam1 was similar in both groups across all time points (Supplemental Figure S8I–K). Interestingly, Apoe expression appeared to increase over time in both groups, although this increase was not statistically significant (Supplemental Figure S8L).

### 3.7. Histological Analysis: VML

**Figure 7:**
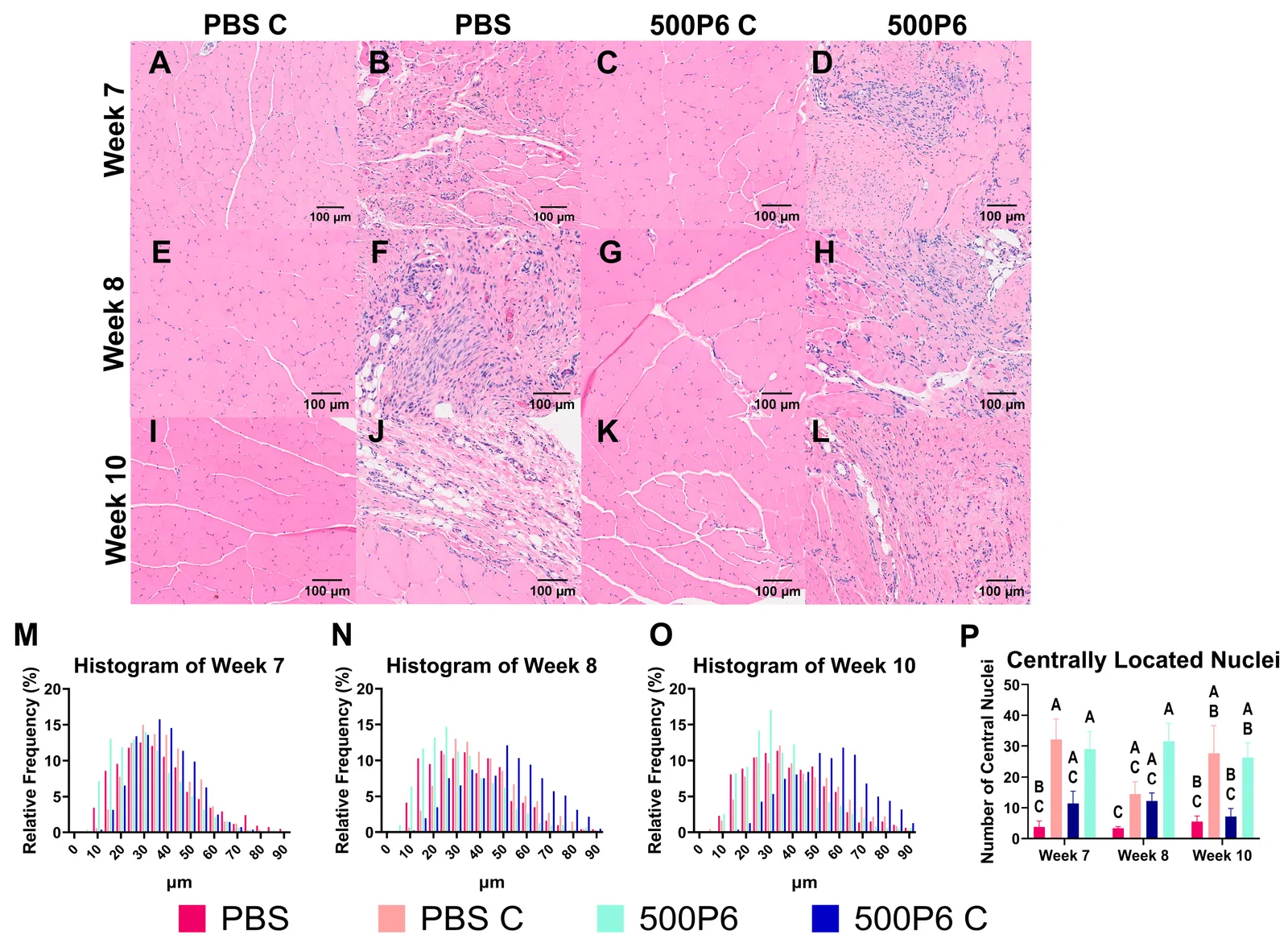
hMDC treatments increase the number of small-diameter fibers in chronic VML injuries and increase the size of muscle fibers in the contralateral limb. Minimum Feret diameter was measured using hematoxylin and eosin-stained sections. Representative sections are shown in (**A**–**L**). Minimum Feret diameter measurements did not change after 1 week of treatment (week 7, (**M**)). Fiber diameters were smaller in cell-treated groups in muscle samples at weeks 8 and 10 (**N**,**O**), while contralateral limbs showed an increase in fiber diameter over the 10-week period (**N**,**O**). Centrally located nuclei were counted, demonstrating an increase in the average number of regenerating fibers in treated limbs (**P**). Groups not sharing a letter are statistically significant with α = 0.05. Data shown are means ± SEM of 8 animals (N = 8).

Figure 7 shows representative images from each group at weeks 7, 8, and 10, stained with hematoxylin and eosin (Figure 7A–L). Feret diameter and centrally located nuclei were quantified from these images. At week 7 (Figure 7M), all groups had a similar average Feret diameter, and the cell-treated group showed a smaller average with a leftward skewness. At week 8, the PBS control group showed an increase in average Feret diameter, while the cell control group showed a more pronounced increase, averaging approximately 50 µm. The cell-treated legs remained skewed to the left with a smaller Feret diameter (Figure 7N). By week 10, the PBS control group showed a similar average Feret diameter to the PBS-treated group, with the cell-treated group having a slightly smaller average. The cell control group again showed an increased average Feret diameter at week 10 (approximately 55 µm), while the cell-treated Feret diameter remained smaller than all other groups (Figure 7O). Centrally located nuclei, a marker of myofiber formation and regeneration, were most abundant in both the PBS and cell treatment groups at week 7 (*p* = 0.0173 and *p* = 0.0066), which correlates with the Feret diameter data. PBS control and cell control groups had significantly fewer centrally located nuclei compared to their respective treatment groups at week 8 and 10 (Figure 7P).

### 3.8. Immunohistochemistry: VML

**Figure 8:**
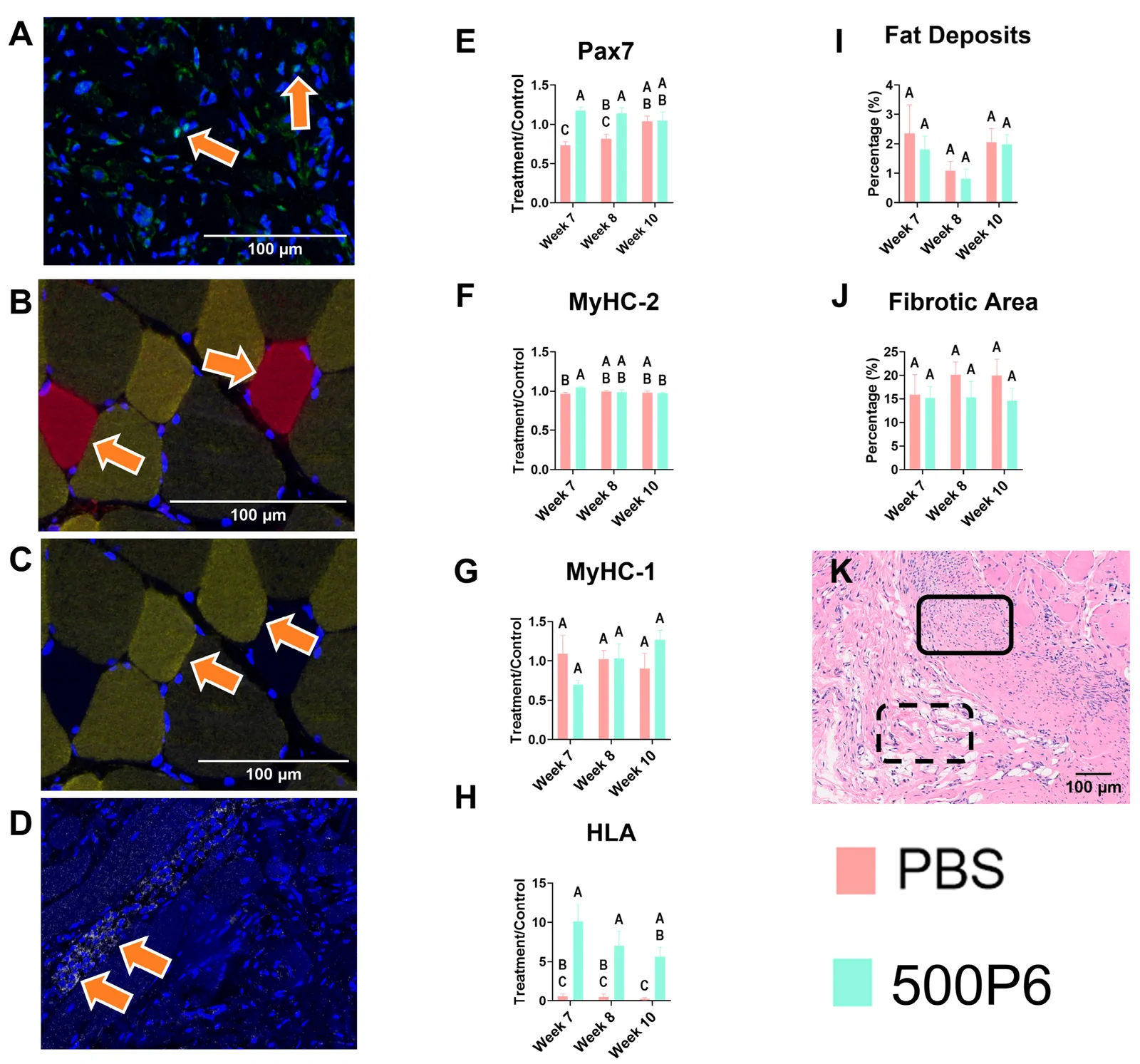
hMDC treatments improve markers of skeletal muscle regeneration in a chronic VML model. Representative images used to quantify Pax7+ counts (**A**), MyHC-1+ fibers (**B**), MyHC-2+ fibers (**C**), and HLA+ cells (**D**). Pax7+ increased in hMDC-treated injury sites at weeks 7 and 8 (**E**). MyHC-2 (fast) fibers increased moderately at week 7 (**F**). MyHC-1 (slow) fibers were unchanged by treatments (**G**). HLA staining showed an increase in the number of HLA+ cells, which was consistent from week 7 to week 10 (**H**). Fatty tissue infiltrate (**I**) and fibrosis (**J**) were unchanged across injury groups using H&E staining. Representative images show fibrotic (solid) versus fat (dashed) tissue (**K**). No HLA-stained fibers were detected in PBS-treated injuries. Groups not sharing a letter are statistically significant with α = 0.05. Data shown are means ± SEM of 8 animals (N = 8).

Immunofluorescence staining was conducted to examine the expression of Pax7 (Figure 8A), MyHC-1 (Figure 8B), MyHC-2 (Figure 8C), and HLA (Figure 8D) with the corresponding quantification shown in Figure 8E–H. In the cell-treated group, Pax7-positive cell levels (green) remained relatively stable across the three time points with significant increases compared to PBS at weeks 7 and 8 (*p* = 0.0007 and *p* = 0.0085). The PBS-treated group showed an increase in Pax7-positive cells from week 7 to week 10 (*p* = 0.0336 Week 7 v Week 10), reaching levels similar to the cell-treated group at week 10 (Figure 8E). MyHC-1-positive fibers (red), representing slow-twitch muscle fibers, showed statistically similar levels in both groups across all time points, although a slight increase was observed in the cell-treated group over time (Figure 8F). Fast-twitch fibers (MyHC-2, yellow) were significantly more abundant in the cell-treated group at week 7 (*p* = 0.0205), but both groups had similar percentages of fast-twitch fibers by weeks 8 and 10 (Figure 8G). HLA staining was significantly higher at all time points in hMDC-treated injuries compared to PBS-treated injuries (*p* < 0.0001, *p* = 0.0066, *p* = 0.0372) (Figure 8H). Notably, no HLA staining was detected in muscle fibers; instead, HLA-positive cells were located in the interstitial spaces (Figure 8D). Fat deposits and fibrotic areas were quantified to assess differences in tissue composition. There were no significant differences in the percentage of fat deposits between groups at any time point, and fat content was generally less than 3% of the total sample area (Figure 8I). Similarly, there were no statistically significant differences in the percentage of fibrotic area between the groups at any time point, indicating that cell treatment did not affect the development of fibrotic tissue (Figure 8J).

## 4. Discussion

Stress urinary incontinence (SUI) is a highly prevalent condition, affecting roughly 20% of individuals over a lifetime and disproportionately impacting women (50% of women > 45 years) [11]. SUI often stems from injury or atrophy of the external urethral sphincter muscle. Since current treatments such as slings and pharmacotherapy do not restore muscle, regenerative approaches using autologous muscle cells have garnered interest. Moreover, Cook Myosite is in phase III clinical trials for its cell therapy treatment using autologous human muscle-derived cells (hMDCs) to treat SUI. In order to better understand hMDC engraftment and its effects on muscle regeneration, we used two muscle injury models, cardiotoxin and VML. We showed that injecting hMDCs into injured muscle markedly enhanced regenerative markers in both acute (cardiotoxin-induced) and chronic (volumetric muscle loss, VML) injury models. These models were chosen to represent mild and severe muscle injuries that might occur in patients with damage to the external urethral sphincter; however, it is recognized that this is also an inherent limitation to the study and preclinical models that directly test the external urethral sphincter are still needed. This is because the structure, size, and immune response of the external urethral sphincter would influence the fibrotic scar that develops in this muscle [14].

In the cardiotoxin (CTX) model, high-dose hMDC treatment (500,000 cells) substantially increased myogenic regulatory factors (Pax7, Myf5, Myog, Myf6) and the fast-twitch muscle fiber marker Myh1 compared to controls. This indicates the activation of muscle stem cells and progression through myogenic differentiation. In the VML model, hMDC delivery preserved Pax7-positive satellite cells over time and transiently increased the fraction of fast-twitch fibers in the regenerating muscle (although these results were not significant). Importantly, hMDC treatment did not exacerbate fibrosis or fat deposition in the injured area. Together, these results demonstrate that hMDC injection can regulate the local environment, shifting it toward a pro-resolving and regenerative environment. This is consistent with the concept that bolstering stem and progenitor cell numbers at injury sites accelerates repair [15], but our study is limited without additional data from a 100,000 p6 injection group.

Our data demonstrated a dose dependency in the regenerative response. Higher cell numbers (500k vs. 100k) consistently produced greater increases in myogenic gene expression and histological markers of regeneration. For example, only the 500k groups showed significant rises in Pax7, Myf5, Myog and Myf6 mRNA after CTX injury, suggesting that a threshold cell dose is required to fully activate the myogenic program. These data are supported by a prior porcine study by Mitterberger et al., who determined that cell doses on the order of 10^7^–10^8^ myoblasts were needed to achieve large improvements in urethral closure pressure, whereas low doses had little effect [16]. Mitterberger followed up this study in humans, demonstrating that 5 × 10^6^ combination of myoblasts and fibroblasts improved electromyography spike amplitude [17]. Surprisingly, cell dose varies widely amongst several studies: 18–22 × 10^6^ [18], 10–50 × 10^6^ [19], 1–50 × 10^6^ [20], 1–128 × 10^6^ [21], 10–200 × 10^6^ [22], 0.6–25 × 10^6^ [23], and 0.2 × 10^6^ [24]. The cell dose used in our study is far lower than the porcine study and lower than most of the doses used in prior studies. Our significant finding demonstrates sufficient evidence that muscle fibers regenerate within the injury site using these lower doses, suggesting that the therapeutic dose response threshold may be lower than what these prior studies tested. In our CTX model, the 500P3 dose groups showed smaller muscle fiber diameters (indicating more newly regenerating fibers), whereas a later-passage group (p6) tended to have fiber sizes closer to uninjured muscle. These findings suggest that early-passage cells may fuse and proliferate rapidly to form thinner fibers initially, while higher-passage cells appeared to either integrate with host fibers and mature or produce chemical factors that promoted fiber maturation.

Interestingly, despite the robust regeneration signals at high dose, this did not translate into immediate force recovery. In fact, the 500P3 group had lower contractile force 7 days post-CTX injury compared to controls. A possible explanation is that delivering higher cell doses initiates a larger-scale remodeling with many small new myofibers and higher cell turnover, temporarily reducing force output until maturation is complete. This phenomenon underscores that greater regenerative activity (higher gene expression, more small fibers) may initially coincide with weaker contractile performance. By contrast, mid-level cell doses or later-passage cells appear to promote larger fibers, with force closer to normal. These data are in contrast to current muscle regeneration dogma, where more muscle regeneration will equate with increased force output. These findings can be contextualized using a recent study by Hoyaux et al., where a sarcomere-based approach was used to model force output [25]. Newly regenerating muscle fibers have a shorter sarcomere length, density, and size compared to mature muscle [26], directly reducing muscle force output. Moreover, Gregorevic et al. highlighted this point by showing that the contractile properties of regenerating fibers were different from their mature counterparts, and those regenerating fibers had approximately 10% of the maximal force-producing capacity of mature fibers [27]. Overall, our results and those of others indicate that cell dosage must be optimized: too few cells may not engraft sufficiently, while too many cells may delay functional recovery by overloading the injury site. We also observed in vitro that early-passage hMDCs formed more myotubes than late-passage cells. While these in vitro results were based on an n = 2 and demonstrate a qualitative finding, they are consistent with a known change in myogenic potential with increased cell expansion. Thus, both cell number and passage history critically influence the balance between rapid muscle regeneration and efficient functional integration.

Optimizing cell transplantation requires an understanding of the cell kinetics, differentiation potential, and fate following engraftment [28]. We demonstrated that hMDCs engrafted into the host tissue using IVIS imaging and HLA staining. Immunohistochemical staining revealed HLA-positive cells alongside HLA-negative fibers, indicating that hMDCs engrafted, proliferated, differentiated, formed fibers, and integrated with the host muscle. We also demonstrated that Pax7 staining increased in response to hMDC engraftment, which was consistent with our gene expression data. We then determined that these increases were associated with HLA (Supplemental Figure S5). Interestingly, HLA-positive fibers were present alongside MyHC-1 staining and not MyHC-2, but this needs to be explored further.

Our findings are in line with preclinical and clinical work on skeletal muscle cell transplantation. In animal models of incontinence, injected myoblasts have been shown to survive, fuse, and improve sphincter function. For example, Chancellor et al. demonstrated that myoblasts injected into a rat urethra/bladder wall remained viable for at least 3–4 days, fused into new myotubes and myofibers in situ, and formed muscle bundles in the smooth muscle layers [29]. Similarly, MDC injections into the urethral wall increased leak point pressure in a rat intrinsic sphincter deficiency model [30]. Mitterberger and colleagues performed landmark human trials in women with SUI. In one study, transurethral injection of autologous myoblasts (with a small number of fibroblasts) cured 79% of patients at 1 year and significantly increased sphincter muscle thickness and contractility [17]. In another, 18 of 20 women were completely continent 1 year after treatment [31]. These clinical results likely reflect the same biology we observe: the injected muscle cells stimulate satellite cell activation and new muscle formation in the sphincter.

Our data extend these prior results by modeling a fibrotic injury (VML) that mimics severe childbirth-related sphincter damage. Indeed, injected cell therapies improved muscle laceration injuries, where human adipose-derived stromal cells increased muscle force, reduced fibrosis, and improved capillary density [32]. Very few studies have investigated the effects of MDC injection in VML or other severe injuries, and no studies were found that injected cell therapy into a chronic muscle injury with significant fibrosis. We determined that even in this scarred environment, hMDC delivery enhanced satellite cell markers and fast-fiber regeneration. Interestingly, cell treatments using the p6 hMDCs increased muscle fiber diameter in the contralateral control muscle. Effects on the contralateral control limb are well-established in orthopedic injuries such as bone fracture [33], observing bone loss in the contralateral limb which is an expected result. In muscle, however, the results are more complex. Immobilization studies show that exercising the non-immobilized limb “cross-trains” the immobilized muscles, suppressing the amount of atrophy [34]. This type of “cross-training” could also apply to a change in circulating cytokines or growth factors such as IGF1 due to MDC injections [35]. The exact mechanism regulating the observed effects in the contralateral limb is unclear at this time, but our data suggest that hMDC therapy may also “cross-train” the contralateral limb, and these results are especially significant when applied to a chronic and challenging fibrotic environment where we did not previously observe an increase in contralateral fiber size [12].

Other stem cell approaches have likewise shown promise for SUI. Fu et al. injected rat adipose-derived stem cells into the urethral musculature of incontinent rats [36]. They reported significant improvements in leak point pressure and increases in urethral muscle thickness and bundle size after 1–3 months [36]. These results, along with ours, support the concept that augmenting sphincter muscle volume and quality with additional myogenic cells can improve continence. Our observation that hMDC injection did not increase fibrosis or fat (both of which could worsen sphincter deficiency) is reassuring and consistent with the safety seen in clinical myoblast transfers.

These findings have several implications for treating female SUI. First, they reinforce that autologous muscle cell injection can be regenerative: by expanding muscle mass and stimulating host satellite cells, it offers a cure-oriented approach rather than mere symptom palliation. Given that SUI affects roughly half of older women, often due to sphincter muscle defects from childbirth, cell therapy may address the root cause. Importantly, our work underscores the need to optimize cell therapy parameters. The dose dependency observed here suggests that therapies should aim for sufficiently high cell numbers. In patients, this might mean harvesting larger muscle biopsies or expanding cells longer (while maintaining potency) to achieve therapeutic doses. Notably, Mitterberger’s porcine data showed >300% pressure increases only at the highest cell concentrations. Clinically, this could translate into improved sphincter closure pressures and reduced leakage, provided the cells engraft well. Second, the results highlight careful consideration of cell quality. We saw that lower-passage cells behaved differently from higher-passage cells. Thus, manufacturing protocols should carefully consider and optimize in vitro “aging” of the cells. Some clinics co-inject fibroblasts or use extracellular matrix scaffolds to support myoblast survival; these strategies might further enhance engraftment in the fibrotic urethral bed. Third, while our molecular and histological data are promising, ultimate benefit in SUI must be measured by continence outcomes (e.g., leak point pressure, pad tests). The lack of functional force improvement in our VML model cautions that muscle regeneration alone may not immediately restore continence. Full functional recovery likely requires not only muscle volume but also proper innervation and coordination. Future preclinical studies should therefore incorporate measures of sphincter function (e.g., urodynamic testing in larger animals). Clinically, combination therapies such as concurrent neuromodulation or electrical stimulation might be considered alongside cell injection.

## 5. Conclusions

Our results highlight the continued development of autologous MDC therapy for SUI. By demonstrating enhanced muscle regeneration in CTX and VML injured tissue with hMDCs, we provide support for the positive outcomes seen in early human trials. These different models also underscore the necessity to study challenging situations to increase treatment success rates in patients. Ultimately, careful optimization of cell dose, passage, and delivery will be critical to maximize the therapeutic potential for this debilitating condition and ensure long-term functional benefits. Future studies should focus on expanding the current VML injection model to include additional passages while also varying the number of cells injected. Future studies should also elucidate the mechanisms underlying the observed differences in regenerative outcomes based on cell characteristics and exploring strategies to enhance myoblast survival, integration, and functional contribution within the context of the complex urogenital environment.

## Figures and Tables

**Table 1:** RNA primers used to detect mRNA levels in gene expression experiments.

**Supplier**	**Gene**	**Temperature (°C)**	**Forward (5′ → 3′)**	**Reverse (3′ → 5′)**
Fisher Scientific	*Apoe*	60	TGTTGGTCCCATTGCTGACAGGAT	TGGTGTTTACCTCGTTGCGGTACT
Fisher Scientific	*Pdgfra*	58	AGTCAGATCCACAGCATCCG	AATGACCGTCCTGGTCTTGC
Fisher Scientific	*Icam1*	61	GACCTGGACACACCTACAGC	TCCCAGAGAGGTCTACGAGC
Fisher Scientific	*Mcp1*	60	AGCCAACTCTCACTGAAGCC	CCTGCTGCTGGTGATTCTCT
Fisher Scientific	*Ctgf*	60	TTTGAGCTTTCTGGCTGCAC	TAATGGCAGGCACAGGTCTT
Fisher Scientific	*Gapdh*	62	ACTCTACCCACGGCAAGTTC	TGGGTTTCCCGTTGATGACC
**Supplier**	**Gene**		**Supplier Cat. #**	
Bio-Rad	*Pax7*	60	PrimePCR™ SYBR^®^ Green Assay: Pax7, Rat desalted 200 × 20 μL reactions UniqueAssayID: qRnoCED0004970 Cat. #10025636	
Bio-Rad	*Myf5*	60	PrimePCR™ SYBR^®^ Green Assay: Myf5, Rat desalted 200 × 20 μL reactions UniqueAssayID: qRnoCED0007849 Cat. #10025636	
Bio-Rad	*Myog*	60	PrimePCR™ SYBR^®^ Green Assay: Myog, Rat desalted 200 × 20 μL reactions UniqueAssayID: qRnoCED0008449 Cat. #10025636	
Bio-Rad	*Myf6*	60	PrimePCR™ SYBR^®^ Green Assay: Myf6, Rat desalted 200 × 20 μL reactions UniqueAssayID: qRnoCED0007939 Cat. #10025636	
Bio-Rad	*Myh1*	60	PrimePCR™ SYBR^®^ Green Assay: Myh1, Rat desalted 200 × 20 μL reactions UniqueAssayID: qRnoCED0051040 Cat. #10025636	
Bio-Rad	*Myh7*	60	PrimePCR™ SYBR^®^ Green Assay: Myh3, Rat desalted 200 × 20 μL reactions UniqueAssayID: qRnoCID0003724 Cat. #10025636	
Bio-Rad	*Itga7*	60	PrimePCR™ SYBR^®^ Green Assay: Itga7, Rat desalted 200 × 20 μL reactions UniqueAssayID: qRnoCID0003101 Cat. #10025636	

**Table 2:** Antibodies used to perform immunohistochemistry.

Primary Antibody	Company (Cat#)	Primary Antibody Dilution	Opal	Opal Dilution	Secondary Antibody Dilution	Antigen Retrieval
HLA Antibody	Cell Signaling (79769)	1/100	520	1/150	1/5 Opal polymer HRP	ER2
PAX7 Polyclonal Antibody	Invitrogen (PA5-23990)	1/100	480	1/150	1/5 Opal polymer HRP	ER1
Polyclonal Rabbit Anti-Mouse Laminin Antibody	LSBio (LS-B4754-0.1)	1/100	620	1/150	1/5 Opal polymer HRP	ER1
Recombinant Anti-Slow Skeletal Myosin Heavy Chain Antibody	Abcam (ab234431)	1/2000	690	1/150	1/5 Opal polymer HRP	ER2
Anti-Fast Myosin Skeletal Heavy Chain Antibody	Abcam (ab91506)	1/1000	TSA-DIG, 570	TSA DIG 1/75 Opal 780 1/50	1/5 Opal polymer HRP	ER1

## Data Availability

The data that support the findings of this study are openly available in FigShare at https://figshare.com/articles/figure/Human_Muscle_Derived_Cells_Injected_into_Acute_and_Chronic_Muscle_Injuries_Engraft_and_Support_Regenerative_Markers/30667097, reference number: 10.6084/m9.figshare.30667097. URL accessed on 25 November 2025.

## Supplementary Materials

The following supporting information can be downloaded at: https://www.mdpi.com/article/10.3390/bioengineering13091022/s1.
