# Supplementary material for: Human Muscle-Derived Cells Injected into Acute and Chronic Muscle Injuries Engraft and Support Regenerative Markers
Source: Bioengineering (Basel). 2026 Sep 2;13(9):1022. doi: 10.3390/bioengineering13091022 (PMC13603626; doi:10.3390/bioengineering13091022)
Supplement: Supplementary file 1 [file bioengineering-13-01022-s001.zip › bioengineering-4513521-supplementary.pdf]

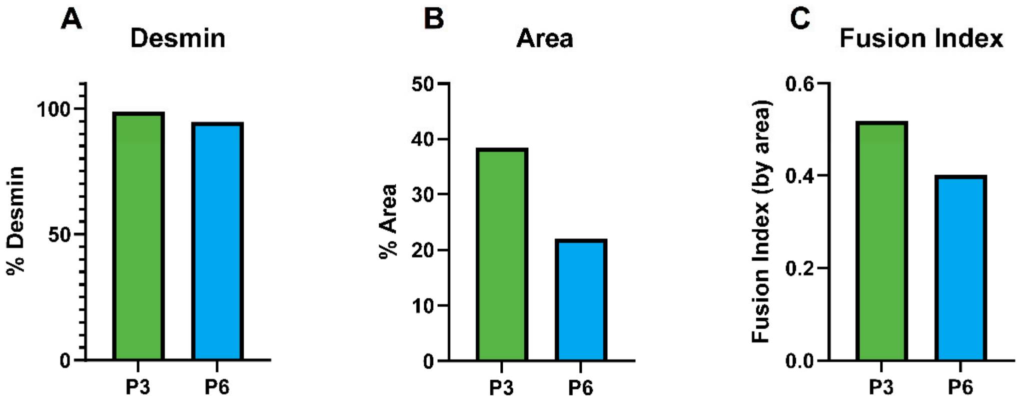

Supplementary Figure S1. Preliminary characterization of hMDCs prior to injection. P3 and P6 hMDCs were cultured *in vitro* and compared using desmin (A), differentiated myotube surface area coverage (B), and fusion index (C). Data shown are means without a SEM due to low sample size (N=2).

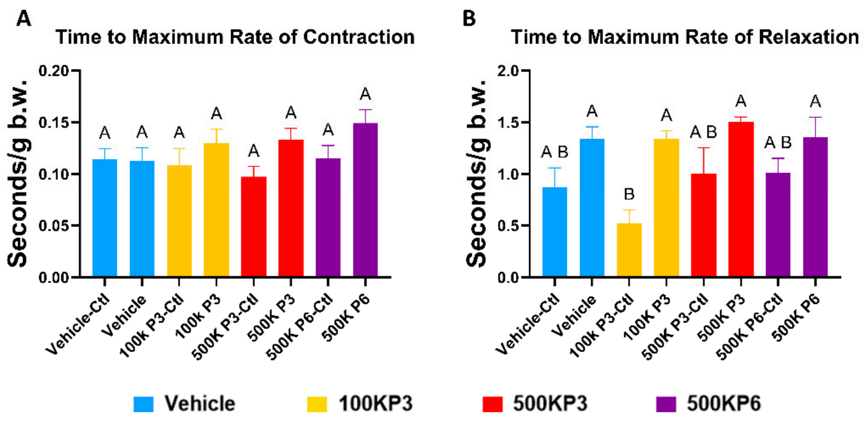

Supplementary Figure S2. Muscle force characteristics. Time to maximum rate of contraction (A) and relaxation (B) were measured. Time to maximum rate of relaxation increased only for the 100P3-treated limbs compared to their contralateral control. Groups not sharing a letter are statistically significant with α=0.05. Data shown are means ± SEM of 8 animals (N=8).

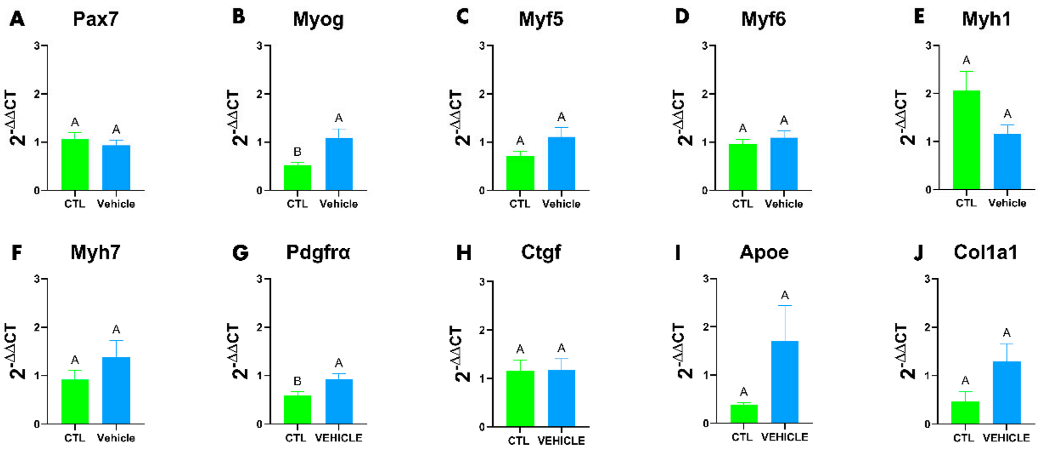

Supplementary Figure S3. mRNA levels for myogenic genes in both contralateral and vehicle control limbs. Pax7 was unchanged (A), Myog (B) was higher in vehicle treated limbs compared to contralateral, Myf5 was unchanged (C), as was Myf6 (D), Myh1 was not different (E), Myh7 was similar between control groups (F), Pdgfra was higher in vehicle treated limbs (G), and Ctgf (H), Apoe (I), Col1a1 (J) were all unchanged. Groups not sharing a letter are statistically significant with α=0.05. Data shown are means ± SEM of 8 animals (N=8).

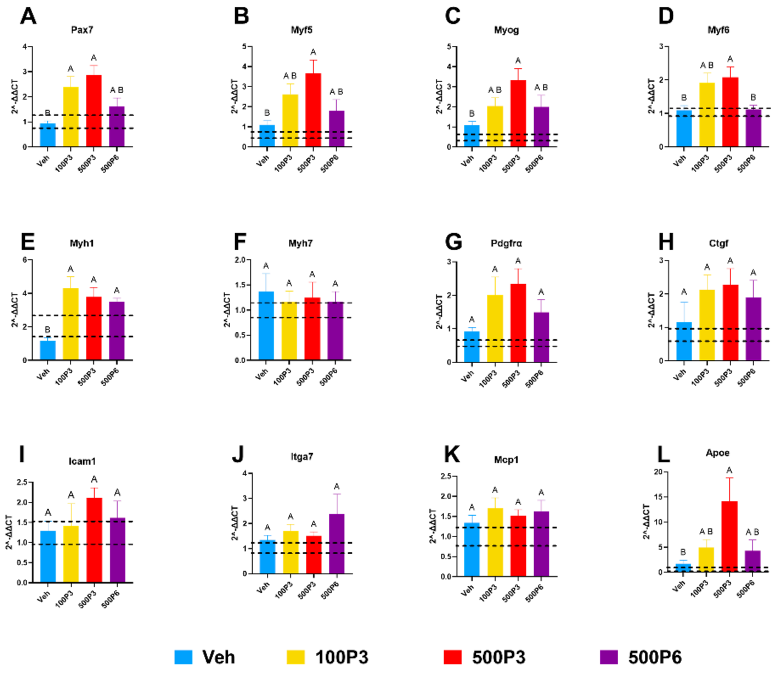

Supplementary Figure S4. $2^{\Delta\Delta CT}$ mRNA levels for myogenic genes. Dotted lines represent the range of contralateral control mRNA values. Pax7 was higher in P3-treated injuries while P6 was similar to both P3 and Vehicle groups (A). Myf5 and Myog were highest in 500P3-treated injuries, while 100P3 and 500P6 were similar (B,C). Myf6 was highest in 500P3-treated injuries, 100P3 was similar to both 500P3 and 500P6, while 500P6 was similar to vehicle (D). Mhy1 was higher in all hMDC-treated injuries (E), while Myh7 was unchanged (F). Pdgfra, Ctgf, Icam1, Itga7, and Mcp1 (G-K) were all unchanged with hMDC treatment. Apoe was highest in 500P3-treated injuries, while 100P3 and 500P6 were similar to 500P3 and to the vehicle (L). Groups not sharing a letter are statistically significant with α=0.05. Data shown are means ± SEM of 8 animals (N=8).

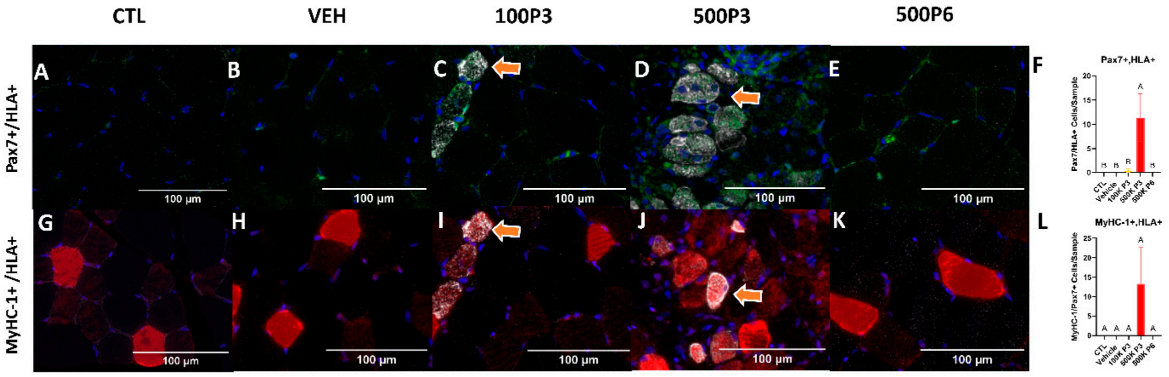

Supplementary Figure S5. OPAL staining shows hMDCs contribute to the Pax7 and MyHC-1 population. Pax7/HLA-stained sections were analyzed and showed the highest number of double-positive cells in 500P3-treated injuries (A-F). MyHC-1/HLA double-positive fibers were stained and showed evidence of double-stained fibers, but statistical significance was not demonstrated due to variability (G-L). Groups not sharing a letter are statistically significant with α=0.05. Data shown are means ± SEM of 8 animals (N=8).

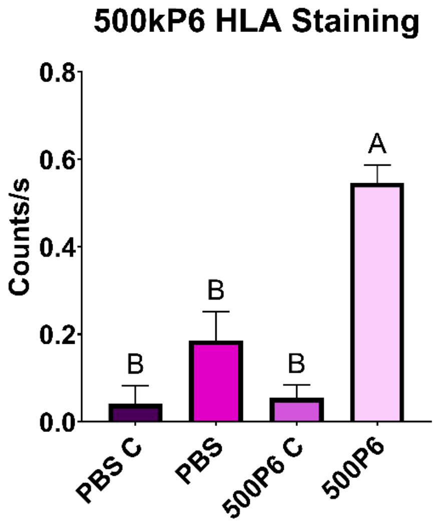

Supplementary Figure S6. hMDCs integrate with host fibers but at much lower intensity levels. HLA staining was analyzed, and determined that 500P6-treated injuries showed elevated intensity levels when compared to PBS treated limbs, PBS contralateral limbs and 500P6 contralateral limbs; however, those numbers were below our threshold when analyzing all treatment groups together.

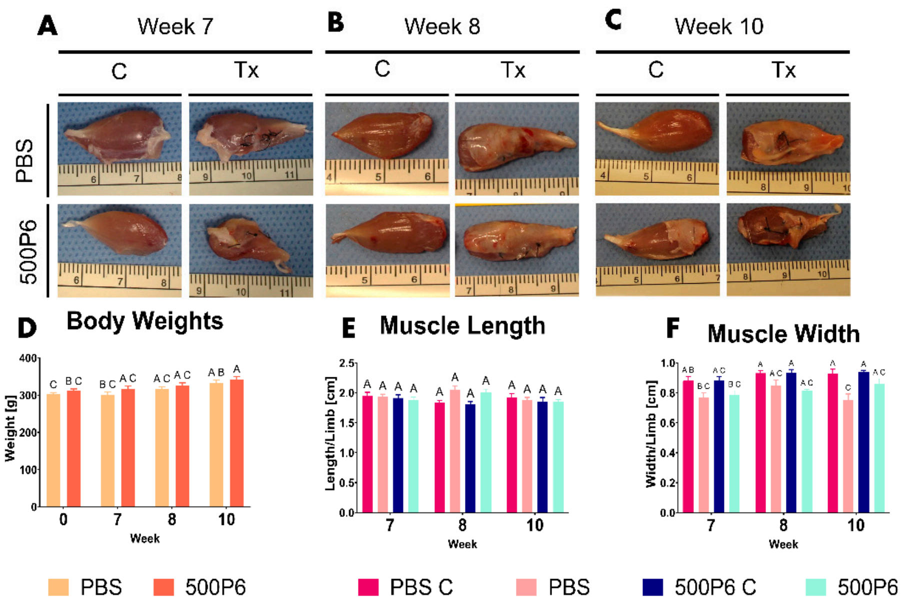

Supplementary Figure S7. Gross morphological evidence demonstrates the extent of the VML injury following treatment. Chronic VML injuries were treated with PBS or hMDCs. Representative images show the different muscles tested (A-C). Animal weight (D), muscle length (E), and muscle width (F) were all measured. Animal weight consistently increased over the duration of the study. Muscle length was unchanged. Muscle width was smaller in PBS-treated limbs compared to hMDC-treated limbs by week 10. Groups not sharing a letter are statistically significant with α=0.05. Data shown are means ± SEM of 8 animals (N=8).

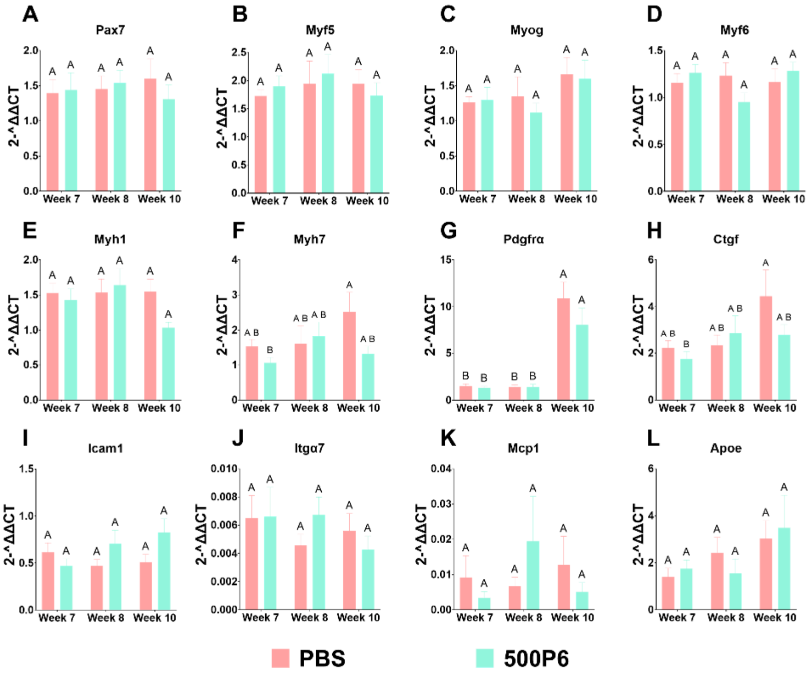

Supplementary Figure S8. mRNA levels following hMDC treatment. Myogenic gene expression was similar for all treatment groups (A-D). Myh1 fast-twitch gene expression was also similar regardless of treatment (E). Myh7 slow-twitch gene expression was elevated by week 10 in PBS-treated injuries, while cell-treated injuries remained unchanged (F). Pdgrfa was elevated by week 10 in both PBS and 500P6 groups (G). Ctgf was elevated by week 10 in PBS-treated injuries, while cell-treated injuries remained unchanged (H). Icam1, Itga7, Mcp1, and Apoe were all unchanged by PBS or cell treatment (I-L). Groups not sharing a letter are statistically significant with α=0.05. Data shown are means ± SEM of 8 animals (N=8).
